# Rapid detection of carbapenemase-producing *Klebsiella pneumoniae* strains derived from blood cultures by Matrix-Assisted Laser Desorption Ionization-Time of Flight Mass Spectrometry (MALDI-TOF MS)

**DOI:** 10.1186/s12866-017-0952-3

**Published:** 2017-03-08

**Authors:** Christina Sakarikou, Marco Ciotti, Camilla Dolfa, Silvia Angeletti, Cartesio Favalli

**Affiliations:** 1 Department of Experimental Medicine and Surgery, “Tor Vergata” University of Rome, Via Montpellier 1, 00133 Rome, Italy; 2 Laboratory of Clinical Microbiology and Virology, Polyclinic “Tor Vergata” Foundation, V.le Oxford 81, 00133 Rome, Italy; 3 Clinical Pathology and Microbiology Laboratory, University Hospital Campus Bio-Medico, Via Alvaro del Portillo 200, 00128 Rome, Italy

**Keywords:** Carbapenemases, Detection, *Klebsiella pneumoniae*, Blood culture, MALDI-TOF MS

## Abstract

**Background:**

Carbapenemase-producing *Enterobacteriaceae* (CPE), particularly carbapenemase-producing *Klebsiella pneumoniae* isolates, are important causative agents of nosocomial infections associated with significant mortality rates mostly in critical wards. The rapid detection and typing of these strains is critical either for surveillance purposes and to prevent outbreaks and optimize antibiotic therapy. In this study, the MALDI-TOF MS method was used to detect rapidly these isolates from blood cultures (BCs) and to obtain proteomic profiles enable to discriminate between carbapenemase-producing and non-carbapenemase-producing strains.

**Results:**

Fifty-five *K. pneumoniae* strains were tested. Identification and carbapenemase-production detection assay using Ertapenem were performed both from bacterial pellets extracted directly from BCs flasks and from subcultures of these strains. For all isolates, a complete antimicrobial susceptibility testing and a genotypic characterization were performed.

We found 100% agreement between the carbapenemase-producing profile generated by MALDI TOF MS and that obtained using conventional methods. The assay detected and discriminated different carbapenemase-producing *K. pneumoniae* isolates within 30 min to 3 h after incubation with Ertapenem.

**Conclusions:**

MALDI-TOF MS is a promising, rapid and economical method for the detection of carbapenemase-producing *K. pneumoniae* strains that could be successfully introduced into the routine diagnostic workflow of clinical microbiology laboratories.

## Background

In recent years, there was a worldwide spread of carbapenemase-producing *Enterobacteriaceae* (CPE), mainly carbapenemase-producing *Klebsiella pneumoniae* isolates, that has become an increasing health care problem all over the world [1–3]. CPE are important etiological agents of hospital-acquired infections (HAI), which cause significant mortality particularly among patients in intensive care units (UCI) [4]. In *Enterobacteriaceae*, carbapenem resistance, (CRE), is mainly induced by two mechanisms: (i) expression of carbapenemases enzymes able to degrade carbapenems; (ii) an association of decreased outer membrane permeability (e.g porin loss or deficiency of porin expression) with overexpression of β-lactamases possessing very weak carbapenemase activity (e.g extended-spectrum β-lactamases ESBLs or acquired AmpC-type cephalosporinases) [5]. From a public health standpoint, carbapenemase producers are taken into major consideration than carbapenem-resistant isolates non carbapenemase producers because their resistance trait can be transferred [6]. The carbapenemase genes transmission by mobile genetic elements leads to a rapid dissemination and carries additional non-β-lactam resistance determinants responsible for resistance to different groups of antibiotics giving rise to multidrug- and pandrug-resistant (MDR, PDR) isolates [7]. Accordingly, a rapid detection of carbapenemases is essential in order to prevent spreading, detect outbreak and for an effective antibiotic therapy to improve the outcome of these patients. However, detection of carbapenemases in Enterobacteria is challenging especially in the presence of carbapenemase-producing *Klebsiella pneumoniae* strains with low carbapenem MICs [8]. For that purpose, the latest molecular- and biochemical-based techniques (e.g RT-PCR, microarray, Carba NP test) have been shown to be suitable to detect early (3–5 h) carbapenemase-producing Enterobacteria directly from positive blood cultures [9–13]. Recently, it has been shown that MALDI-TOF MS could represent an alternative to detect rapidly CPE strains as well as to identify rapidly the pathogens [14–17]. As mass spectrometry measures mass differences, a MALDI-TOF MS-based assay was performed to detect the hydrolysis of different β-lactam antibiotics after a short co-incubation of the Gram-negative isolate with the specific antibiotic. Therefore, hydrolyzed and non-hydrolyzed substances which differ in their molecular weights could be monitoring [15, 17].

In this study, 55 consecutive non-replicated clinical strains of *K. pneumoniae*, isolated from hospitalized patients with Gram-negative bacteremia were tested. The study focused on carbapenemase-producing *Klebsiella pneumoniae* strains because of their increasing prevalence in Italian hospitals [18]. The identification and the carbapenemase-production detection assay using Ertapenem were performed from both bacterial pellets extracted directly from BCs flasks and from subcultures of these isolated strains.

## Methods

### Specimens collection

A total of 55 consecutive non-replicated clinical strains of *K. pneumoniae*, isolated from blood cultures of hospitalized patients, were collected from July 2014 to July 2015 in two hospitals of Rome, Italy. In particular, 47 were isolated at the Microbiology Laboratory of Polyclinic “Tor Vergata” Foundation and 8 at the Clinical Pathology and Microbiology Laboratory of the University Hospital “Campus Bio-Medico”.

The blood specimens were collected in BD BACTEC™ Plus Aerobic/F and BD BACTEC™ Plus Anaerobic/F vials and incubated in a Bactec FX blood culture system (Becton Dickinson, NJ, USA). Clinical and epidemiological data of patients were also recorded. The isolates were collected for surveillance and public health purposes, as done routinely in clinical setting, and no ethical approval was required.

### Sample preparation

At the Microbiology laboratory of Polyclinic “Tor Vergata” Foundation, a total of 170 consecutive positive BCs containing Gram-negative rods were included in this study. After the BC was flagged as positive, Gram staining was performed. In the presence of Gram-negative rods, MALDI-TOF MS identification was performed directly from the bacterial pellet extracted from the BC flasks, using the MALDI Sepsityper kit (Bruker Daltonics, Bremen, Germany), according to the manufacturer’s instructions, in an autoflex speed mass spectrometer (Bruker Daltonics, Bremen, Germany). Moreover, an additional bacterial pellet was extracted for the carbapenemase-production detection assay.

For the identification assay, an ethanol-formic acid extraction was performed on the bacterial pellets obtained using the Sepsityper kit (pellets contained 10^7^ to 10^9^ CFU), according to manufacturer’s instructions.

In the Clinical Pathology and Microbiology laboratory of the University Hospital “Campus Bio-Medico”, only the MALDI-TOF MS identification was performed directly from the bacterial pellet extracted from the BC flaks using the MALDI Sepsityper kit (Bruker Daltonics, Bremen, Germany) as described above, on a Microflex LT mass spectrometer (Bruker Daltonics, Bremen, Germany). However, the carbapenemase-production detection assay for the same isolates was performed only from subcultures of frozen strains at the Microbiology laboratory of Polyclinic “Tor Vergata” Foundation on an autoflex speed mass spectrometer (Bruker Daltonics, Bremen, Germany).

### MALDI-TOF MS-based carbapenemase-production detection assay

The bacterial pellets obtained using the Sepsityper kit (pellets contained 10^7^ to 10^9^ CFU) were resuspended in 30 μl of Ertapenem solution (1 mg/mL in 1 mM ammonium hydrogen citrate buffer pH 7.1) and incubated for 3 h at 37 °C under agitation for the carbapenemase-production detection assay. Incubated suspensions with Ertapenem solution were centrifuged at 13.000 rpm for 2 min, and 1 μl of the supernatant was spotted in quadruplicate onto a polished steel target plate MTP 384 and air-dried spots were overlaid with 1 μl of HCCA matrix in organic solvent. Data are collected at 30-min intervals during the entire incubation time. All measurements were performed on an autoflex speed mass spectrometer (Bruker Daltonics, Bremen, Germany), within a mass range of 100 to 1.100 Da, while the spectra were manually examined using the FlexAnalysis 3.4 software (Bruker Daltonics, Bremen, Germany). The spectra were acquired in the linear positive mode at a laser frequency of 200 Hz. A method optimized for the low mass range was set up using the following parameters: acquisition range, 100 to 1,000 Da with maximum laser frequency; acceleration voltage, IS1 voltage 19.66 kV; IS2 voltage, 18.51 kV; lens 6.99 kV and extraction delay time, 350 ns. For instrument calibration, an external standard solution consisting of bradykinin 1–7 (757.40 Da), 2 HCCA peaks (CCA_[M + H]^+^ 190.05 Da; and CCA_[2 M + H]^+^ 379.02 Da) and angiotensin (1049, 54) (Bruker Daltonics, Bremen, Germany) (100–1000 Da range) was used. A reference wild-type strain of *K. pneumoniae* was used as a negative control while a KPC-producing *K. pneumoniae* reference strain was used as a positive control and both of them were included in each run.

For quality control of the antibiotic solution and for detection of spontaneous hydrolysis a sample with Ertapenem solution only, without bacteria, was processed as all the other samples.

The obtained spectra were not reported in this section because they are periodically deleted for room reasons from the computer used for the routine laboratory analyses.

### Subcultures processing

Identification and the carbapenemase-production detection assay were also performed from subcultures of the strains isolated on MacConkey agar, Columbia blood agar and Chocolate PolyViteX plates (BioMèrieux, France) after 18–24 h incubation at 37 °C. After overnight incubation, 30 μl of Ertapenem solution was inoculated separately with a 1 μl-inoculation loop filled with both single bacteria colonies isolated from overnight cultures and control strains and incubated for 3 h at 37 °C under agitation for the carbapenemase-production detection assay. For the identification assay, a small part of an isolate’s colony was inoculated on the polished steel target plate MTP 384 and overlaid with 1 μl of HCCA matrix. In the same manner, the respective measurements were performed as described above.

### Strain characterization

For all *K. pneumoniae* isolates, a complete antimicrobial susceptibility testing was created from isolates cultured onto chocolate PolyViteX agar after overnight incubation at 37 °C using the Vitek 2 automated system (bioMérieux, France) and the AST cards N-202 for a phenotypic characterization. The MIC values were interpreted according to EUCAST breakpoints tables 2014. A genotypic characterization for the resistance genes *bla*_KPC_, *bla*_NDM_, *bla*_OXA-48,_ *bla*_VIM_, *bla*_IMP_, *bla*_SHV_, *bla*_TEM_, *bla*_CTX-M_,was performed by Polymerase Chain Reaction (PCR) (Qiagen, Hilden, Germany), using primers already reported in literature [11, 17]. The DNA was extracted from each isolated strain by EZI Advanced XL automated system (Qiagen, Hilden, Germany).

## Results

### MALDI-TOF MS identification performance

At the Microbiology laboratory of Polyclinic “Tor Vergata” Foundation, 175 isolates, tested directly from blood culture samples, were correctly identified at the species level by MALDI-TOF MS; there was a 100% agreement with the subculture MALDI-TOF MS-based identification routinely performed in our laboratory. One-hundred and forty-six isolates fell into the *Enterobacteriaceae* family whereas 29 were non fermentative Gram-negatives rods. Among the *Enterobacteriaceae* isolates, 47 were identified as *K. pneumoniae* strains. All identifications exhibited a Biotyper score value of > 2.2. Moreover, the identifications obtained directly from blood culture samples had a score values >2.3 compared with the score obtained from subculture. Data, using the direct method, were obtained earlier respect to the routine method: 15 min after the BC was flagged as positive versus 12 h of the routine method.

### MALDI-TOF MS-based carbapenemase-production detection assay performance

**Fig. 1:**
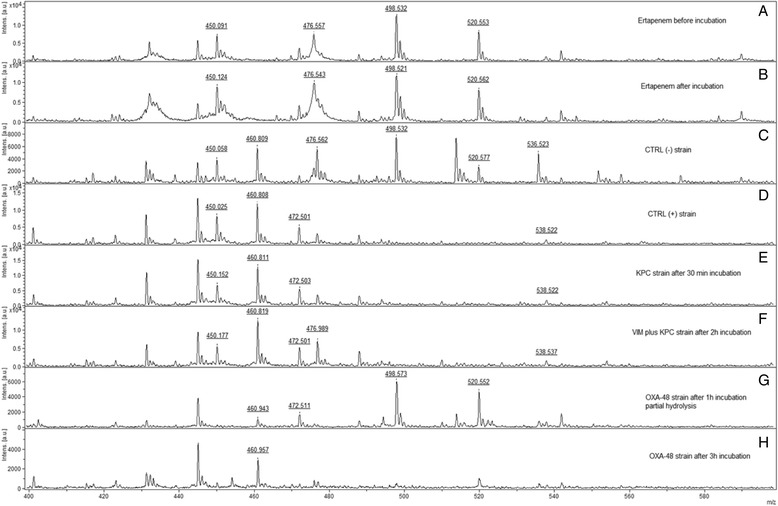
Mass Spectra of non-hydrolyzed and hydrolyzed Ertapenem and mass spectra of Ertapenem degradation after incubation with KPC, KPC plus VIM and OXA-48 producing *K. pneumonia*e strains at different time points, respectively. Molecular weight values of Ertapenem and its salt adducts peaks are indicated at the top of the peaks

Data were collected at 30-min intervals during the entire incubation time. Differences in the hydrolysis rate were easily detected. In the presence of sensitive *K. pneumoniae* strains, intact Ertapenem displayed the following peak pattern: 476.5 Da, 498.5 Da, 520.5 Da, corresponding to the molecular peak of Ertapenem, its single sodium adduct and its double sodium adduct, respectively. These peaks were not found in the spectra obtained from carbapenemase-producing strains. Instead, a mass peak was found at 472.5 Da corresponding to the hydrolyzed decarboxylated sodium adduct while another peak was found at 538.5 Da corresponding to the hydrolyzed double sodium adduct [19]. A m/z 450 Da peak was also detectable in all spectra. Ertapenem only was also incubated up to 3 h as quality control and no spontaneous hydrolysis was seen. Moreover, no difference was observed when the carbapenemase-production detection assay was performed either on solid media growth isolates or isolates extracted directly from BCs. Thus, sensitivities and specificities were 100% for both subcultures and blood cultures. Furthermore, the assay revealed and discriminated KPC-producing strains from other carbapenemases after a 30 min incubation with Ertapenem (Fig. 1). Thus, KPC- plus VIM- producing strains were detected after 2 h incubation with Ertapemen while OXA-48-producing strains were detected after 3 h incubation with Ertapenem. However, after 1 h incubation a partial hydrolysis of Ertapenem was observed in the presence of OXA-48 strains as indicated by the loss of its molecular peak in the presence of the peaks of its salt adducts.

Carbapenemase production was detected by MALDI-TOF MS, and these results were compared with those achieved by routine methods, MIC determination and PCR analysis. No discordance was observed in terms of bacteria classification according to resistance profile (Table 1). Of the 55 isolates investigated, 40 were found to be carbapenemase-producing strains and 15 non- carbapenemase-producing strains.

**Table 1:** Description of carbapenemase-producing *K. pneumoniae* strains used in the carbapenemase-production detection assay

	MIC Ertapenem (mg/l)	MIC Imipenem (mg/l)	MIC Meropenem (mg/l)	Hydrolysis of Ertapenem	Time point of hydrolysis (h)
31 *K.pneumoniae* KPC	≥8	≥16	≥16	+	0.5 h
5 *K.pneumoniae* KPC,VIM	≥8	≥16	≥16	+	2 h
4 *K.pneumoniae* OXA-48	≥8	≤2	≤2	+	3 h

Considering the time to results, the report of the MALDI-TOF-based assay was available after 3 h the BCs were flagged as positive. Instead, 12 to 24 h were necessary using the routine AST, and 5 h by PCR assay. So, the time saved for generating the report ranged from 11 to 21 h comparing MALDI-TOF *vs* routine AST and 2 h comparing MALDI-TOF with PCR assay.

## Discussion

Antibiotic resistance in CPE is a life-threatening condition, so a prompt diagnosis is crucial for improving patient’s prognosis, choose the optimal antibiotic therapy and implement surveillance network. In this context, molecular typing helps to define the extent of the outbreak, identify the source of infection and the reservoir [20, 21], while proteomic profiling may represent a valid alternative to biochemical and genome-based identification and susceptibility detection assays.

In this study, we examine the performance of MALDI-TOF MS identification assay and the mass spectrometric carbapenemase-production detection assay directly on positive BC flasks from hospitalized patients with Gram-negative bacteremia. The MALDI-TOF MS technology appeared accurate, cost saving and rapid.

It was investigated the Ertapenem hydrolysis induced by different carbapenemase-producing *K. pneumoniae* strains isolated from positive blood cultures. Using 1 mL of blood culture fluid, the obtained amount of bacteria producing carbapenemase was sufficient to hydrolyze the antibiotic in a time frame ranging from 30 min to 3 h of incubation. The different peak patterns derived from carbapenemase and non-carbapenemase-producing strains allowed the unequivocal identification of these strains. All spectra displayed a mass peak at 450 Da corresponding to the hydrolyzed and decarboxylated forms of Ertapenem. Likely, this peak reflects an unspecific signal derived from a slight background hydrolysis. Similarly, the mass peak at 460 Da detected in all spectra, except those of Ertapenem solution alone, is probably another unspecific signal derived from the blood culture medium [19]. For these reasons, these two peaks were not classified among the spectra originated from BCs (Fig. 1).

A characteristic peak pattern that allowed the discrimination between the carbapenemase-producing and non- carbapenemase-producing plated bacteria was also obtained with the bacteria derived directly from fresh positive BCs. Therefore, it can be envisaged that the growth medium does not affect significantly the activity of the carbapenemase enzymes.

The method presented in this work does not require particularly skilled personnel, and may be used in any laboratory equipped with a MALDI-TOF spectrometer.

In this study, we decided to set up the assay on Ertapenem hydrolysis as it is associated with a characteristic peak pattern easily detected using only the organic matrix HCCA which is used also for bacteria identification. Furthermore, Ertapenem is considered a good candidate for carbapenemase producers detection as reported by Nordmann et al. 2012 [6, 19].

For the 55 K. *pneumoniae* strains studied, no false-negative results were found. This is important, because the aim of the assay is to obtain a rapid information for a timely and effective antibiotic therapy.

Therefore, the test showed a high sensitivity and this is important for an appropriate antibiotic stewardship, as a good sensitivity allows a higher level of confidence in the administration of narrow-spectrum antibiotics in presence of susceptible isolates [17].

Furthermore, carbapenemase-production detection assay was able to detect and discriminate between different carbapenemase-producing *K. pneumoniae* isolates within 30 min to 3 h after incubation with Ertapenem. The rapid detection of carbapenemase-producing bacteria from positive BCs, combined with a proactive antimicrobial stewardship program, is important because the choice of the optimal therapy is crucial for the prognosis of patients with sepsis. With the present protocol, a report regarding the effectiveness of carbapenem therapy is obtainable within a few hours after the BC is flagged positive. The presence/absence of characteristic peak patterns resulting from carbapenemase-producing and non-carbapenemase-producing strains has been used as the cut-off of the assay with maximum sensitivity and specificity.

The MALDI-TOF MS-based assay and the approved routine method provided the same classification results. Furthermore, identification and carbapenemase-production detection testing by MALDI-TOF MS can be performed in parallel, as widely the same, and the additional hands-on time required is moderate.

To evaluate whether potential residues of blood culture fluid could interfere with the reproducibility of the measurements, the analysis was performed in quadruplicate. No interference was observed.

**Fig. 2:**
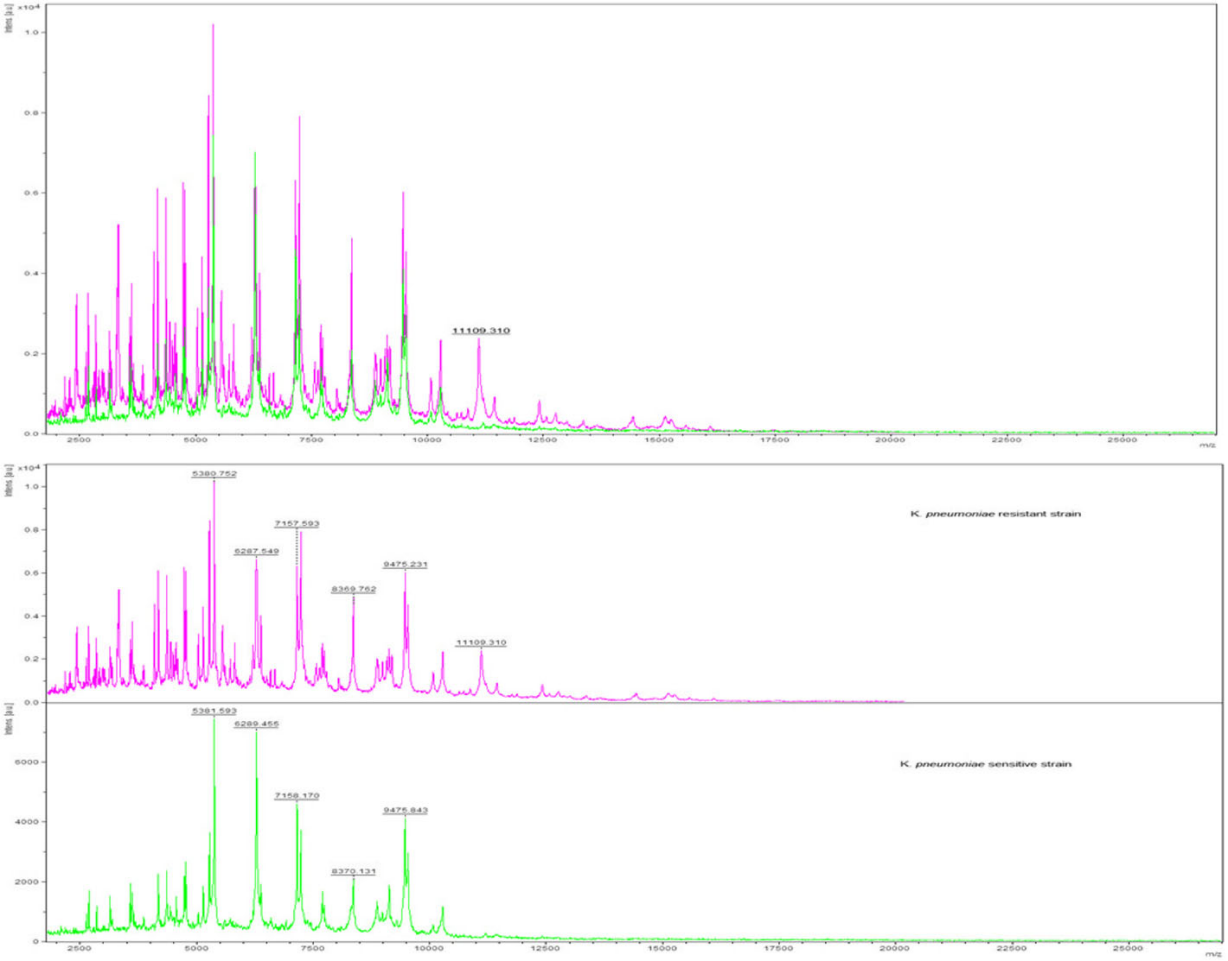
Identification of a MALDI-TOF MS peak associated with *bla*_KPC_. Presence of the 11,109-Da peak in KPC-carrying *K. pneumoniae* isolate mass spectrum (fuchsia) and absence of an 11,109-Da peak in a *K. pneumoniae* non KPC-carrying isolate mass spectrum (*green*), respectively. This specific peak could be a helpful real-time marker in epidemic strain detection in a hospital setting

All *bla*_KPC_ positive isolates showed a peak within the window of 11,109 ± 15 Da that was absent in all *bla*_KPC_ negative isolates of *K. pneumoniae* (Fig. 2). This peak has been suggested by Lau et al. (2014) as a *bla*_KPC_-containing pKpQIL plasmid-identifying proteomic marker that could be implemented in the routine screening of the clinical laboratory workflow and may monitoring epidemiological dissemination mapping in real time during any future CRE hospital outbreak [22]. These data suggest a monoclonal spreading of all KPC-producing *K. pneumoniae* isolates in our hospitals, probably correlated to the hyper-epidemic clonal complex (CC) 258 (ST-258 producing KPC-2 or KPC-3, and ST-512 producing KPC-3) which was considered as the predominant clone in Italy [18]. The positive detection of ~ 11,109 Da mass peak associated with *bla*_KPC_-containing pKpQIL plasmid could be a real-time marker of carbapenem-producing strains of great clinical and epidemiological value as suggested by Lau et al. (2014) [22]. It could be easily implemented in the routine diagnostic activity of any microbiology laboratory as part of the organism identification by MALDI-TOF MS without placing additional constraints on laboratory resources. All these findings confirm the capacity of MALDI-TOF MS technology to identify timely a carbapenemase producer causing a bacteremia. Compared with the molecular-based techniques the cost saving is significant and no particular skilled personnel is required [21]. In addition, a proteomic approach unlike a genomic provides information on gene expression without focusing on single genes. Indeed, genomic assays may not reveal novel carbapenemase genes as the range of resistance genes to be detected is predefined.

The MALDI-TOF MS carbapenemase-production detection assay could be used to confirm carbapenemase activity either in routine and reference laboratories and might supplement other rapid and cost-effective tests used in carbapenemase detection such as the phenotypic Carba NP test.

However, these preliminary findings requires further assessment and confirmation, preferably by multicenter validation studies in order to avoid clonal bias as well as analytical error and develop universal guidelines for data interpretation.

## Conclusion

In summary, the presented carbapenemase-producing strain detection by MALDI-TOF MS technology could improve the workflow of BCs positive to *Enterobacteriaceae* strains which show a suspicious susceptibility profile, and enhance the stewardship of carbapenems by revising the resistance surveillance program at different levels.

A final same-day report will be available to clinicians and to hospital epidemiologists improving patient management and treatment; thus, preventing outbreaks and spreading of pathogens in the hospital with beneficial effects on the health care system.

Compared to other alternative approaches, like molecular-genetic methods that remain costly with practical limitations, this method is cost effective, highly sensitivity and easy to perform.

However, a single diagnostic technology cannot be universally applicable in clinical microbiology as the detection of a limited number of specific determinants cannot accomplish the complex issue of antibiotic resistance.

## Abbreviations

AmpC: Class C β-lactamases
AST: Antimicrobial Susceptibility Test
BC: Blood culture
CC 258: Clonal complex 258
CPE: Carbapenemase-producing *Enterobacteriaceae*
CRE: Carbapenem-resistant *Enterobacteriaceae*
CTX-M: Cefotaximase
ESBLs: extended-spectrum b-lactamases
EUCAST: European Committee on Antimicrobial Susceptibility testing
HAI: Hospital-Acquired infection
HCCA: α-cyano-4-hydroxy cinnamic acid
ICU: Intensive-care unit
IMP: Imipenemase
KPC: *Klebsiella Pneumoniae* Carbapenemase
MALDI-TOF MS: Matrix-assisted laser desorption ionization-time of flight mass spectrometry
MDR: Multidrug resistant
MIC: Minimum inhibitory concentration
NDM: New Delhi Metallo-beta-lactamase
OXA-48: Oxacillinase-48
PCR: Polymerase chain reaction
PDR: Pandrug resistant
SHV: Sulfhydryl variable
ST 258: Sequence type 258
TAT: Turn around time
TEM: A β-lactamase named after a Greek patient Temoneira
VIM: Verona Integron-encoded Metallo-beta-lactamase
